# Effect of the Herbal Drug Guilu Erxian Jiao on Muscle Strength, Articular Pain, and Disability in Elderly Men with Knee Osteoarthritis

**DOI:** 10.1155/2014/297458

**Published:** 2014-09-16

**Authors:** Chen-Chen Tsai, Yin-Yi Chou, Yi-Ming Chen, Yih-Jing Tang, Hui-Ching Ho, Der-Yuan Chen

**Affiliations:** ^1^Department of Traditional Chinese Medicine, Taichung Veterans General Hospital, 1650 Taiwan Boulevard, Section 4, Taichung, 40705, Taiwan; ^2^College of Chinese Medicine, China Medical University, No. 91, Hsueh-Shih Road, Taichung, Taiwan 40402, Taiwan; ^3^Hungkuang University, No. 1018, Section 6, Taiwan Boulevard, Shalu District, Taichung 43302, Taiwan; ^4^Division of Allergy, Immunology and Rheumatology, Department of Internal Medicine, Taichung Veterans General Hospital, 1650 Taiwan Boulevard, Section 4, Taichung 40705, Taiwan; ^5^Faculty of Medicine, National Yang Ming University, No. 155, Section 2, Linong Street, Taipei, 112, Taiwan; ^6^Department of Family Medicine, Center for Geriatrics and Gerontology, Taichung Veterans General Hospital 1650 Taiwan Boulevard, Section 4, Taichung 40705, Taiwan; ^7^School of Medicine, Chung Shan Medical University, No. 110, Section 1, Jianguo North Road,Taichung 40201, Taiwan; ^8^Biostatistics Task Force of Taichung Veterans General Hospital, 1650 Taiwan Boulevard, Section 4, Taichung 40705, Taiwan; ^9^Institute of Biomedical Science, National Chung Hsing University, 250 Kuo Kuang Road, Taichung 402, Taiwan

## Abstract

*Background*. Guilu Erxian Jiao (GEJ) is a widely used Chinese herbal remedy for knee osteoarthritis, but its clinical efficacy is unknown. *Methods*. We enrolled 42 elderly male patients with knee OA, including 21 patients who received the herbal drug GEJ as the case group and 21 patients who did not receive GEJ as the control group. The effects of 12 weeks of GEJ treatment on muscle strength of lower limbs were measured by a Biodex dynamometer, with disability evaluated on the Lequesne index and articular pain measured on the visual analog scale (VAS) between the two groups on the baseline and after treatment. *Results*. There were significant increases in the levels of muscle strength of TQ/BW-ext-dominant and TQ/BW-flex-dominant between the two groups after treatment (*P* < 0.05). There were also significant increases in muscle strength of knee extensor muscles in the GEJ-treated group (*n* = 21) self-controlled before and after 12 weeks of treatment (all P < 0.01). There were significant decreases in articular pain (P < 0.01) and Lequesne index scores (P < 0.01) in the GEJ-treated group when compared to the non-GEJ-treated group. *Conclusions*. Our results showed that GEJ is effective and is tolerated well in elderly men with knee OA.

## 1. Introduction 

Knee osteoarthritis (OA) is a common articular disease, affecting not only the joints but also the surrounding muscles and causing falls, disabilities, and dependency in older people [1, 2]. Quadricep strength deficits have been reported in 20%–70% of patients with knee OA [3, 4]. Any improvement in muscle strength or peak power of the lower extremities with decreased levels of articular pain may be important and is a strong predictor of functional ability [5–7].

There is no gold standard treatment for knee OA. Pharmacological approaches include analgesics, anti-inflammatory agents, intra-articular corticosteroids or hyaluronic acid, glucosamine sulphate, chondroitin sulphate, and some experimental treatments already widely used. However, these drugs have some adverse effects such as constipation, nausea, and excessive sedation in older people, and their effect on cartilage with OA symptoms remains controversial [8–15].

The herbal drug Guilu Erxian Jiao (GEJ) is a multicomponent Chinese herbal supplement that has been used for treatment of degenerative joint diseases without adverse effects for two thousand years [16–20]. However, understanding of the mechanisms responsible for the beneficial effects of GEJ is limited. Specifically, information on the effects of GEJ on muscle strength, articular pain, and disability in elderly men with knee osteoarthritis is scant. Therefore, it is very important to investigate the clinical effectiveness and safety of GEJ therapy for elderly men with knee OA.

The primary purpose of the present study was to investigate the therapeutic effects of 12 weeks of GEJ treatment on muscle strength in elderly male patients with knee OA. The secondary purpose was to investigate the effects of the same regimen on articular pain, the Lequesne disability index, and blood markers (liver and renal function).

## 2. Materials and Methods

### 2.1. Patients

A total of forty-two elderly men with knee OA who were regularly followed up at outpatient clinics of the departments of rheumatology and traditional Chinese medicine (TCM) were enrolled. Patients were included if they met the following criteria: males aged 65 years or older who could walk independently, fulfilled the 1986 American College of Rheumatology (ACR) classification of knee OA [21], received the same Western medication for OA regularly for 24 weeks before the study entry, had persistent articular pain and disability while under regular Western medication, and regular 12 weeks followed up during the study enrolled by a physician of rheumatology. Patients who had a history of coronary artery disease, previous knee surgery, severe visual impairment, stroke, severe pulmonary disease requiring the use of oxygen, hip fracture or lower extremity joint replacement in the past 6 months, and other inflammatory and/or infectious diseases, or who used other Chinese medicine or health products as indicated in their medical records, were excluded. The case group consisted of twenty-one patients who regularly received GEJ treatment (6 g/daily) for 12 weeks simultaneously and were followed up at the outpatient clinic of the department of TCM. The control group consisted of 21 patients who do not receive GEJ treatment and only continued to receive the same Western medication at the outpatient clinic of the department of rheumatology that had been prescribed for them before the study entry. Patient characteristics and variables, which were recorded at baseline and after treatment, included the following: age, BMI, Lequesne index, visual analog pain scale score (VAS), muscle power, liver function, and renal function. The study was approved by the Institutional Review Board (IRB) of Taichung Veterans General Hospital, Taiwan. A departmental database was searched to identify elderly patients with OA who were treated with GEJ between March 2007 and March 2008. The Ethics Committee of Taichung Veterans General Hospital approved this study (C06287) and the written consent of each participant was obtained according to the Declaration of Helsinki.

### 2.2. Study Design and Protocol

We enrolled 42 elderly men with knee OA, including 21 patients who received the herbal drug GEJ as the case group and 21 patients who did not receive GEJ and received the same Western medication as the control group. This study evaluated the effects of 12 weeks of GEJ treatment on muscle strength of lower limbs, physical function, and articular pain. The dominant side of the OA knee was defined as the joint with articular pain (VAS) > 5.

Twenty-one patients received four capsules three times a day of Guilu Erxian Jiao extract, 6 g/daily for 12 weeks. The GEJ capsules (batch number: CB118-091610) were prepared according to the well-documented TCM formula described in a TCM book known as “The Golden Mirror of Medicine,” and provided by the Sun Ten Pharmaceutical Company, Ltd., Taiwan (drug permit license number-042004, Department of Health, Taiwan). The Sun Ten Pharmaceutical Company, Ltd. has received the herbal Good Manufacturing Practice (GMP) certification in Taiwan. The components of the GEJ capsules are Carapax and Plastrum Chrysemys (species:* Pseuclemys scripta elegans*,* Chrysemys scripta elegans*; Animal part: Plastrum); Cornu Cervi (species:* Rangifer tarandus*; animal part: antler); Ginseng Radix Rubra (species:* Panax ginseng* C. A. Mey; plant part: root) and* Lycii fructus* (species:* Lycium barbarum L.*; plant part: fruit). GEJ was prepared as follows: Carapax, Plastrum Chrysemys, and Cornu Cervi were stewed for 7 days, and then Ginseng Radix Rubra and Lycii Fructus were added into the mixture of Carapax, Plastrum Chrysemys, and Cornu Cervi [20]. A 2.8 g extract was derived from the ratio between the 4 components consisting of about 5 g (Carapax and Plastrum Chrysemys); 10 g (Cornu Cervi); 1.1 g (Ginseng Radix Rubra); 0.9 g (Lycii Fructus). Each 6 g/daily capsule contained 2.8 g extract and 3.2 g cornstarch in conformance to the drug permit license.

### 2.3. Measurement of Muscle Strength

Muscle strength of the knee extensors and flexors was assessed by an isokinetic dynamometer (Biodex Medical Systems, Inc., Shirley, NY, USA) according to the peak torque/body mass variable (Nm/kg) at a velocity of 60°/s. Maximum strength tests (one repetition maximum or 1RM) were performed fortnightly. Then, a new 80% load was prescribed during the interval between the strength tests. Participants were given 3% increments in load per session as tolerated [22, 23]. The calculation of muscle performance and the respective normalizations for body mass were performed by using the dynamometer software.

### 2.4. Measurement of Articular Pain

The pain severity of knee OA was evaluated by the visual analogue scale (VAS) at baseline and after 12 weeks in the two groups on a weight-bearing posture (walking) for 5 minutes. The instrument consists of horizontal lines 10 cm long, with anchor points of 0 (indicates no pain) and 10 (indicates maximum degree of pain.). The average of three measurements was recorded, and a 10-minute interval was set between tests to allow for more consistent measurement conditions [24].

### 2.5. Measurement of OA Disability

Disability of patients with knee OA was evaluated using the Lequesne index (LI) [25]. The questionnaire is divided into three parts: (I) pain or discomfort (5 questions), (II) maximum distance walked (2 questions), and (III) activities of daily living (4 questions). A score of 26 (maximum) indicates the greatest degree of dysfunction, and a score of 1–3 (minimum) indicates mild dysfunction [25, 26].

### 2.6. Statistical Analysis

Descriptive statistics were run on all variables. Associations among variables, including baseline characteristics, muscle strength, the Lequesne index, and pain outcomes, were evaluated with the Mann-Whitney *U* test. Incremental and decremental changes in quantitative variables after 12 weeks of treatment were compared with baseline data in the non-GEJ-treated group and the GEJ-treated group. The level of statistical significance was set at 0.05 for all hypothesis testing. When significant changes in the levels of muscle strength and decremental changes of VAS scores and Lequesne index scores between the two groups pre- and posttreatment were found, the variables of self-controlled were evaluated using the Wilcoxon signed-rank test in the GEJ-treated group before and after 12 weeks of treatment. Statistical significance for all tests was accepted with *P* values less than 0.05. A sample size calculation indicated that statistical significance could be achieved with 84% power using sample power 2.0 software.

We used logistic regression to estimate the ORs and 95% CIs for the association between the GEJ-treated group and the non-GEJ-treated group. Effects of herbal drug treatment were also assessed and the results were analyzed to identify any trends. The level of significance was established at *P* < 0.05 (tested by Wald statistic). The logistic regression was adjusted additionally for incremental changes of torque/body weight extension of the dominant and nondominant side (TQ/BW-ext-dominant and nondominant side); torque/body weight extension of the dominant side after treatment (TQ/BW-ext-dominant-side after treatment); torque/body weight flexion of the dominant side after treatment (TQ/BW-flex-dominant side after treatment); and decremental changes of subcategories in the Lequesne index: (I) change of pain or discomfort, (II) change of maximum distance walked, and (III) change of activities of daily living.

## 3. Results

### 3.1. Clinical Characteristics of OA Patients

Table 1 shows the baseline clinical characteristics of OA patients. There were no significant differences in age, body mass index (BMI), activities of daily living (ADL) scores, Lequesne index scores, balance tests, and muscle strength between the GEJ-treated group and the non-GEJ-treated group.

### 3.2. Changes in Muscle Strength after 12 Weeks of GEJ Treatment

**Figure 1:**
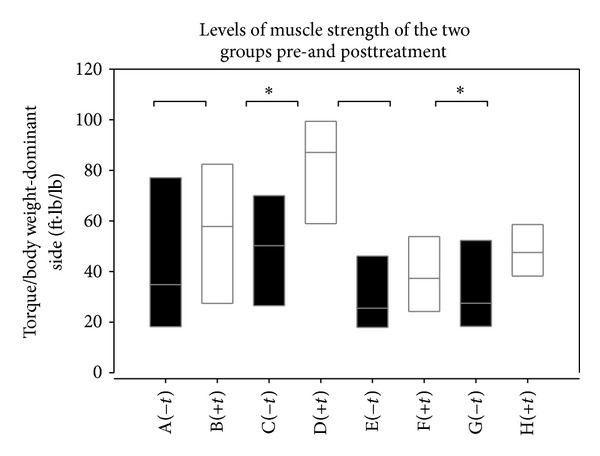
Box plots showing Torque/body weight of extension and flexion-dominant side for each subgroup. A (−*t*) group, TQ/BW-ext-pretreatment in Non-GEJ-treated group; B (+*t*) group, TQ/BW-ext-pretreatment in GEJ-treated group; C (−*t*) group, TQ/BW-ext-posttreatment in non-GEJ-treated group; D (+*t*) group, TQ/BW-ext-posttreatment in GEJ-treated group; E (−*t*) group, TQ/BW-flex-pretreatment in non-GEJ-treated group; F (+*t*) group, TQ/BW-flex-pretreatment in GEJ-treated group; G (−*t*) group, TQ/BW-flex-posttreatment in non-GEJ-treated group; H (+*t*) group, TQ/BW-flex-posttreatment in GEJ-treated group.

Table 2 shows there were no significant incremental changes in the muscle strength of extensor and flexor muscles between the GEJ-treated group and the non-GEJ-treated group from baseline to the end of the 12 week GEJ treatment period (*P* > 0.05, Table 2). Nevertheless, there were slightly higher incremental changes in the strength of TQ/BW-ext-nondominant and TQ/BW-ext-dominant extensor muscles (*P* < 0.1, Table 2) in the GEJ-treated group compared to those in the non-GEJ-treated group. There were no significant differences in the levels of TQ/BW-ext-dominant and TQ/BW-flex-dominant extensor muscles between the two groups at baseline (*P* > 0.05, Table 2). But there were significant increases in the levels of strength of TQ/BW-ext-dominant and TQ/BW-flex-dominant muscles in the GEJ-treated group compared to those in the non-GEJ-treated group (*P* < 0.05, Table 2 and Figure 1). Moreover, we also found that there were significant increases in strength of knee extensor muscles on the dominant side and nondominant side in the GEJ-treated group (*n* = 21) self-controlled before and after 12 weeks of treatment (all *P* < 0.01, Table 3 and Figure 3). Logistic regression analysis demonstrated a trend toward increase of extensor muscle strength on the dominant side (OR = 1.04; 95% CI 1.00–1.09; *P* = 0.062; Table 4).

### 3.3. Changes in Visual Analog Pain Scale Scores (VAS) after 12 Weeks of GEJ Treatment

**Figure 2:**
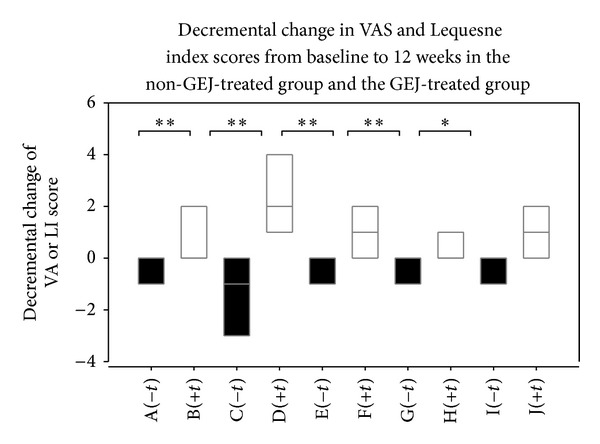
Box plots showing decremental change of VA or LI score for each subgroup. A (−*t*) group, decremental change of visual analog pain scale score in non-GEJ-treated group; B (+*t*) group, decremental change of visual analog pain scale score in GEJ-treated group; C (−*t*) group, decremental change of Lequesne index score in non-GEJ-treated group; D (+*t*) group, decremental change of Lequesne index score in GEJ-treated group; E (−*t*) group, (I) change of pain or discomfort in non-GEJ-treated group; F (+*t*) group, (I) change of pain or discomfort in GEJ-treated group; G (−*t*) group, (II) change of maximum distance walked in non-GEJ-treated group; H (+*t*) group, (II) change of maximum distance walked in GEJ-treated group; I (−*t*) group, (III) change of activities of daily living in non-GEJ-treated group; J (+*t*) group, (III) change of activities of daily living in GEJ-treated group.

**Figure 3:**
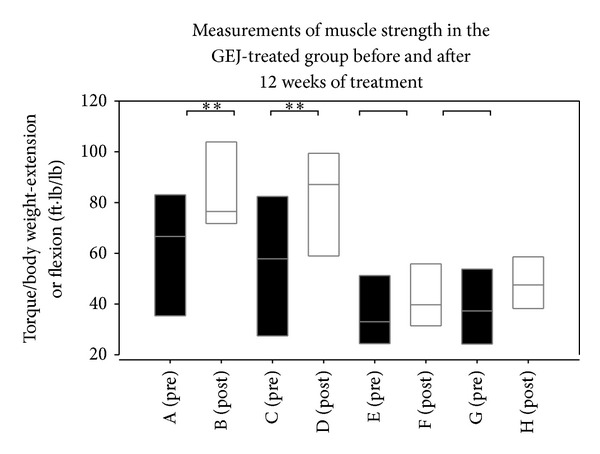
Box plots showing Torque/body weight of extension or flexion for each subgroup. A (pre) group, TQ/BW-ext-nondominant at pretreatment; B (post) group, TQ/BW-ext-nondominant at posttreatment; C (pre) group, TQ/BW-ext-dominant at pretreatment; D (post) group, TQ/BW-ext-dominant at posttreatment; E (pre) group, TQ/BW-flex-nondominant at pretreatment; F (post) group, TQ/BW-flex-nondominant at posttreatment; G (pre) group, TQ/BW-flex-dominant at pretreatment; H (post) group, TQ/BW-flex-dominant at posttreatment.

**Figure 4:**
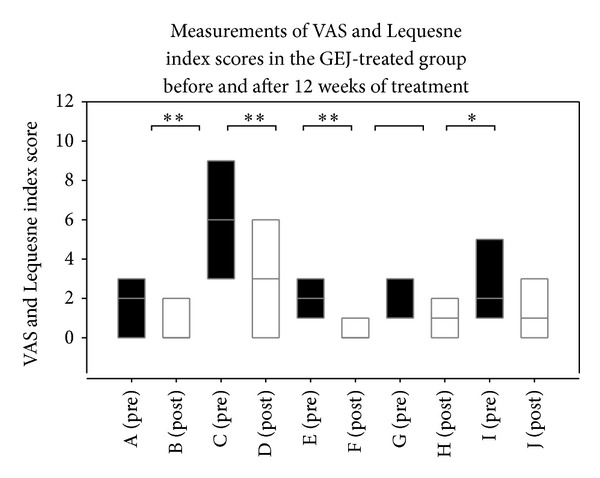
Box plots showing VAS and Lequesne index score for each subgroup. A (pre) group, VAS at pretreatment; B (post) group, VAS at posttreatment; C (pre) group, Lequesne index score at pretreatment; D (post) group, Lequesne index score at posttreatment; E (pre) group, (I) pain or discomfort at pretreatment; F (post) group, (I) pain or discomfort at posttreatment; G (pre) group, (II) maximum distance walked at pretreatment; H (post) group, (II) maximum distance walked at posttreatment; I (pre) group, (III) activities of daily living at pretreatment; J (post) group, (III) activities of daily living at posttreatment.

Table 2 shows that there was a significantly greater decrease in articular pain scores in GEJ-treated patients than in non-GEJ-treated patients (*P* < 0.01) (Figure 2). Table 3 also shows that there was a significant decrease in articular pain scores in the GEJ-treated group before and after 12 weeks of treatment (*P* < 0.01) (Figure 4). Logistic regression analysis also demonstrated a significant decrease in articular pain scores after 12 weeks of GEJ therapy (OR = 2.33; 95% CI 1.09–4.98; *P* = 0.029; Table 4).

### 3.4. Changes in Lequesne Index Scores after 12 Weeks of GEJ Treatment

Table 2 shows there was a significantly greater decrease in Lequesne index (LI) scores of disability in GEJ-treated patients than in non-GEJ-treated patients (*P* < 0.01) (Figure 2). GEJ-treated patients also had a significant reduction in scores in all three parts of the Lequesne index: (I) pain or discomfort (*P* < 0.01), (II) maximum distance walked (*P* < 0.01), and (III) activities of daily living (*P* < 0.05). Table 3 shows there was a significant decrease in LI scores of disability observed in the GEJ-treated group before and after 12 weeks of treatment (*P* < 0.05). GEJ-treated patients also had a significant reduction in scores in two parts of the LI: (I) pain or discomfort (*P* < 0.01) and (III) activities of daily living (*P* < 0.05) in the GEJ-treated group before and after 12 weeks of treatment (Figure 4). Logistic regression analysis demonstrated a significant decrease in one component of the LI, (III) activities of daily living, after 12-weeks of GEJ therapy (OR = 2.28; 95% CI 1.12–4.65; *P* = 0.023) (Table 4).

### 3.5. Changes in Liver and Renal Function after 12 Weeks of GEJ Treatment

The levels of glutamic oxaloacetic transaminase (GOT) and glutamic pyruvic transaminase (GPT) were within normal limits after 12 weeks of GEJ treatment. There were no significant changes in renal function and urinalysis or urinary albumin-to-creatinine ratio (ACR = urine microalbumin/urine creatinine) after 12 weeks of GEJ treatment (*P* > 0.1). No cardiopulmonary dysfunction or adverse gastrointestinal effects were observed during the treatment period.

## 4. Discussion 

To the best of our knowledge, the present study is the first attempt to investigate the therapeutic effects of the herbal drug GEJ on muscle strength, articular pain, and disability (Lequesne index) in elderly men with knee OA. Our results showed that elderly men displayed significant improvements in the muscle strength of the knee and a decrease of articular pain and Lequesne index scores after 12 weeks of GEJ treatment. All patients tolerated the therapy well and reported no adverse effects or changes in liver or renal function.

Scientific data regarding the improvement of muscle strength after GEJ treatment in elderly men with knee OA are scant. In traditional Chinese medicine, GEJ has been used to treat kidney deficiency (qi, ying, and yang) and nourish blood, which plays an important role in treating joint disease at the cellular level [18, 20, 27]. The present study demonstrated a significant increase in muscle strength ofextensor and flexor muscles after 12 weeks of GEJ treatment in elderly men with knee OA, with the most significant increase in the TQ/BW-ext-dominant. Improvement of muscle strength and balance and relief of articular pain are very important for elderly men with knee osteoarthritis who are prone to falls and may become disabled [3, 4]. GEJ may improve muscle strength and walking ability, which may in turn help these patients maintain functional independence [6, 28, 29]. Further studies are needed to elucidate the action mechanism of GEJ for improvement of muscle strength in elderly men with knee osteoarthritis.

Our results also showed a significant decrease in articular pain scores in GEJ-treated patients compared to non-GEJ-treated patients. An in vitro study revealed that GEJ could inhibit the apoptosis of chondrocyte-like phenotype bone mesenchymal stem cells (BMSCs), which reduced synovitis and blocked cartilage destruction [30]. In mice, some results may be related to mechanisms regarding the chondroprotective effect of GEJ achieved through upregulation of bcl-2 mRNA expression and downregulation of caspase-3mRNA expression [18, 30]. In healthy volunteers, the results of a recent randomized, placebo-controlled, double-blind clinical trial study showed that the reversibility of the CD4+ to CD8+ lymphocyte ratio in the GEJ-treated group may explain the effectiveness of GEJ for treatment of knee OA [20]. No clinical changes in biochemical indicators of liver function, renal function, or hemograms were observed after GEJ treatment [20]. These results may explain the mechanism of the therapeutic effects of the herbal drug GEJ on articular pain. A previous study evaluated the effect of over-the-counter osteophyte capsules, which contain extracts of Radix Rehmannia* Rehamanniae* (Dihuang) and Herba Cistanches (Roucongrong), a traditional Chinese herb used for treatment of inflammation and articular pain in osteoarthritis. However, that study was limited by lack of placebo control and there was no double-blinding [31]. Another study investigated the effects of blood-nourishing and hard-softening (BNHS) capsules, a traditional Chinese formula used in the symptomatic treatment of inflammation and pain. BNHS capsules consist of extracts from Bai Shao (*Radix Paeoniae Alba*), Qin Jiao (*Radix Gentianae Macrophyllae*), and Gan Cao (*Radix Glycyrrhizae*). This herbal drug can relieve disease-related symptoms within 4 weeks, such as pain and stiffness, as well as improving physical function in patients with painful knee OA [32]. In a clinical trial, Duhuo Jisheng Wan (DJW-herbal remedy) demonstrated clinical efficacy when compared to diclofenac after 4-weeks treatment. Only in the 4 weeks of treatment were the mean changes in VAS of the DJW-treated group significantly lower than those of the diclofenac-treated group. Afterwards, these mean changes became no different throughout the study [33]. The variables that may have influenced the results of the aforementioned studies are the age of patient populations, the severity of knee OA, the duration of medication taken, and the effectiveness of drugs used. The diverse results and lack of correlation in these studies may be due to several factors, including patient anxiety, motivation, habit of exercise, muscle atrophy, and aberrant joint mechanics [34]. In the present study, elderly men (mean age: 82-83 years) with knee OA who previously had poor compliance experienced a significant decrease in articular pain.

Moreover, there were decremental changes in all components of the Lequesne index in the GEJ-treated group compared to the non-GEJ-treated group after 12 weeks of treatment. In the GEJ-treated group, there was a significant reduction in two parts of the Lequesne index: (I) pain or discomfort and (III) activities of daily living. Our results were similar to the findings of some studies showing the efficacy of individualized Chinese herbal medication, which according to TCM diagnosis can reduce symptoms of OA. The results demonstrated statistically significant improvement in global WOMAC scores and WOMAC subscale scores (for pain, stiffness, and functional impairment) for knee OA [35]. It has previously been shown that patients with OA have impaired muscle activation compared with healthy controls and that pain in OA is assumed to be a major source of inhibition in the ability to voluntarily activate muscles surrounding arthritic joints [3, 36]. In the present study, the pain reduction after 12 weeks of GEJ treatment may have led to improved muscle activation in these patients. So, it may be related to increasing activities of daily living after having improvement on muscle strength and articular pain with GEJ treated. However, the exact mechanism of GEJ's effect on Lequesne index scores remains unclear.

Our results showed no significant influence of GEJ on liver or renal function, suggesting that it had no harmful impact on the biochemical profiles of patients. In a previous study, no adverse gastrointestinal effects, such as abdominal fullness, constipation, diarrhea, or nausea, were observed during 8 weeks of GEJ therapy [20]. If long-term ingestion of GEJ is feasible in the further treatment of elderly men with knee OA, we suggest regular follow-up of liver and kidney function via blood tests for a better understanding of the mechanisms of drug side effects and interactions.

The limitations of our study included the small sample size, the enrollment of men only, the short follow-up period, the advanced stage of knee OA, and the failure to measure life quality. It is possible that the statistical power of our study may not have been sufficient to detect potential differences between the two groups. Some patients might not have taken GEJ regularly during the study period. Some important confounding variables were also not fully addressed, such as diet or dietary supplements, which are known to affect articular pain. This may explain why there were only a few differences in muscle strength between the GEJ-treated group and the non-GEJ-treated group. It is possible that our selection criteria were too stringent. Patients who had a history of coronary artery disease, previous knee surgery, severe visual impairment, stroke, severe pulmonary disease requiring the use of oxygen, hip fracture or lower extremity joint replacement in the past 6 months, and other inflammatory and/or infectious diseases, or who used other Chinese medicine or health products as indicated in their medical records, were excluded. This makes it difficult to transfer the results to the general population with knee OA. However, the stringent selection criteria were necessary to ensure the homogeneity of the groups and, furthermore, to exclude people who were at high risk of having side effects. Furthermore, the elderly people with OA knee treated by GEJ are likely to be taking other drugs, such as analgesics or anti-inflammatory agents, so caution had to be exercised to avoid drug interactions that might lead to other medical problems.

## 5. Conclusions

Our results showed a significant improvement of the muscle strength of the lower limbs, above all the extensor muscle on the dominant side, in elderly men with OA knee who received 12 weeks of GEJ treatment. There were also significant decreases of articular pain scores and Lequesne index scores in these patients. GEJ treatment was well tolerated and caused no significant changes in liver or renal function. An improvement in physical function is clinically important in OA patients because it is a crucial predictor of the ability to perform daily activities and avoid falls. Future clinical trials will be required to extend and confirm these findings and a long-term follow-up is necessary to verify whether the benefits can be maintained.

## Figures and Tables

**Table 1:** Baseline clinical characteristics of the non-GEJ-treated group and the GEJ-treated group.

Variables	Non-GEJ-treated group (*n* = 21)			GEJ-treated group (*n* = 21)			*P* value
	Median	*P*_25_	*P*_75_	Median	*P*_25_	*P*_75_	
Age, years	83.00	81.00	87.00	82.00	80.00	86.00	0.528
BMI, kg/m^2^	24.39	23.11	25.16	23.44	21.23	25.79	0.462
ADL	100.00	100.00	100.00	100.00	100.00	100.00	0.075
Lequesne index	4.00	0.00	6.00	6.00	3.00	9.00	0.108
Test for balance	0.00	0.00	0.00	0.00	0.00	0.00	0.317
PeakTQ-ext-nondominant (Nm)	31.60	21.50	52.90	38.80	18.20	45.90	0.725
PeakTQ-ext-dominant (Nm)	25.70	12.00	52.50	29.10	19.50	49.60	0.624
PeakTQ-flex-nondominant (Nm)	17.40	11.60	26.70	19.50	14.40	32.60	0.801
PeakTQ-flex-dominant (Nm)	17.30	10.80	28.90	18.80	15.70	33.20	0.345
TQ/BW-ext-nondominant (ft*·*lb/lb)	43.10	29.60	93.10	66.60	35.30	83.10	0.538
TQ/BW-ext-dominant (ft*·*lb/lb)	34.80	18.20	77.10	57.80	27.40	82.40	0.365
TQ/BW-flex-nondominant (ft*·*lb/lb)	26.30	16.40	40.20	33.00	24.40	51.20	0.473
TQ/BW-flex-dominant (ft*·*lb/lb)	25.50	17.90	46.10	37.30	24.20	53.80	0.204

Mann-Whitney *U* test; ∗*P* < 0.05.

**Table 2:** Changes in muscle strength, VAS, and Lequesne index scores from baseline to 12 weeks in the non-GEJ-treated group and the GEJ-treated group.

Variables	Non-GEJ-treated group (*n* = 21)			GEJ-treated group (*n* = 21)			*P*-value
	Median	*P*_25_	*P*_75_	Median	*P*_25_	*P*_75_	
Incremental changes in muscle strength							
PeakTQ-ext-nondominant (Nm)	2.10	−4.80	14.30	17.70	3.90	28.20	0.090
PeakTQ-ext-dominant (Nm)	4.60	−1.60	11.70	14.80	3.30	23.20	0.105
PeakTQ-flex-nondominant (Nm)	3.30	−3.40	8.20	2.90	−1.20	10.20	0.308
PeakTQ-flex-dominant (Nm)	3.70	−1.40	8.80	2.80	−2.40	10.60	0.870
TQ/BW-ext-nondominant (ft*·*lb/lb)	4.70	−2.60	26.60	29.70	6.90	51.30	0.080
TQ/BW-ext-dominant (ft*·*lb/lb)	6.10	−2.90	20.40	29.30	6.00	37.30	0.063
TQ/BW-flex-nondominant (ft*·*lb/lb)	5.20	−6.00	11.60	4.90	−1.80	17.90	0.308
TQ/BW-flex-dominant (ft*·*lb/lb)	6.20	−2.50	12.70	4.80	−3.70	16.40	0.811
Levels of muscle strength of the two groups pre- and posttreatment							
TQ/BW-ext-dominant-pretreatment (ft*·*lb/lb)	34.80	18.20	77.10	57.80	27.40	82.40	0.365
TQ/BW-ext-dominant-posttreatment (ft*·*lb/lb)	50.20	26.50	70.00	87.10	58.90	99.40	0.012∗
TQ/BW-flex-dominant-pretreatment (ft*·*lb/lb)	25.50	17.90	46.10	37.30	24.20	53.80	0.204
TQ/BW-flex-dominant-posttreatment (ft*·*lb/lb)	27.50	18.30	52.30	47.50	38.20	58.60	0.045∗
Decremental change of visual analog pain scale score	0.00	−1.00	0.00	0.00	0.00	2.00	0.001∗∗
Decremental change of Lequesne index score	−1.00	−3.00	0.00	2.00	1.00	4.00	0.0003∗∗
(I) change of pain or discomfort	0.00	−1.00	0.00	1.00	0.00	2.00	0.002∗∗
(II) change of maximum distance walked	0.00	−1.00	0.00	0.00	0.00	1.00	0.003∗∗
(III) change of activities of daily living	0.00	−1.00	0.00	1.00	0.00	2.00	0.025∗

Mann-Whitney *U* test; ∗*P* < 0.05, ∗∗*P* < 0.01.

**Table 3:** Measurements of muscle strength, VAS, and Lequesne index scores in the GEJ-treated group (*n* = 21) before and after 12 weeks of treatment.

Variables	Pretreatment			Posttreatment			
	Median	*P*_25_	*P*_75_	Median	*P*_25_	*P*_75_	*P* value
Measurements of muscle strength							
PeakTQ-ext-nondominant (Nm)	38.80	18.20	45.90	52.10	37.90	65.30	0.007∗∗
PeakTQ-ext-dominant (Nm)	29.10	19.50	49.60	51.60	40.10	62.60	0.001∗∗
PeakTQ-flex-nondominant(Nm)	19.50	14.40	32.60	20.70	19.10	36.20	0.085
PeakTQ-flex-dominant (Nm)	18.80	15.70	33.20	30.80	21.70	36.70	0.092
TQ/BW-ext-nondominant (ft*·*lb/lb)	66.60	35.30	83.10	76.50	71.70	103.90	0.006∗∗
TQ/BW-ext-dominant (ft*·*lb/lb)	57.80	27.40	82.40	87.10	58.90	99.40	0.001∗∗
TQ/BW-flex-nondominant (ft*·*lb/lb)	33.00	24.40	51.20	39.70	31.40	55.80	0.092
TQ/BW-flex-dominant (ft*·*lb/lb)	37.30	24.20	53.80	47.50	38.20	58.60	0.076
Measurements of visual analog pain scale score (VAS)							
visual analog pain scale score	2.00	0.00	3.00	0.00	0.00	2.00	0.005∗∗
Measurements of Lequesne index score							
Lequesne index score	6.00	3.00	9.00	3.00	0.00	6.00	0.001∗∗
(I) Pain or discomfort	2.00	1.00	3.00	0.00	0.00	1.00	0.003∗∗
(II) Maximum distance walked	1.00	1.00	3.00	1.00	0.00	2.00	0.150
(III) Activities of daily living	2.00	1.00	5.00	1.00	0.00	3.00	0.032∗

Wilcoxon signed-rank test; ∗*P* < 0.05; ∗∗*P* < 0.01.

**Table 4:** Logistic regression analysis of the changes in the non-GEJ-treated group (*n* = 21) and the GEJ-treated group (*n* = 21).

Variable	Regression coefficient	S.E.	Odds ratio	95% CI of odds ratio	*P* value
Incremental change of TQ/BW-ext-dominant	0.041	0.022	1.04	1.00–1.09	0.062
Decremental change of visual analog pain scale score	0.846	0.388	2.33	1.09–4.98	0.029∗
Change of (III) activities of daily living score	0.824	0.364	2.28	1.12–4.65	0.023∗
Constant	−1.262	0.616	0.28		0.040

*P < 0.05 (tested by Wald statistic).

Accuracy of model = 78.6%.

Model III: backward regression.

## List of Abbreviations

BMI: Body mass index
ADL: Activities of daily living
Peak TQ-ext-nondominant: Peak torque extension of nondominant side
Peak TQ-ext-dominant: Peak torque extension of dominant side
Peak TQ-flex-nondominant: Peak torque flexion of nondominant side
Peak TQ-flex-dominant: Peak torque flexion of dominant side
TQ/BW-ext-nondominant: Torque/body weight extension of nondominant side
TQ/BW-ext-dominant: Torque/body weight extension of dominant side
TQ/BW-flex-nondominant: Torque/body weight flexion of nondominant side
TQ/BW-flex-dominant: Torque/body weight flexion of dominant side
VAS: Visual analogue scale.
